# A Rare Devastating Complication of Lasik: Bilateral Fungal Keratitis

**DOI:** 10.1155/2010/450230

**Published:** 2010-11-11

**Authors:** H. Taylan Sekeroglu, E. Erdem, K. Yar, M. Yağmur, T. R. Ersoz, A. Uguz

**Affiliations:** ^1^Department of Ophthalmology, Cukurova University, Yuregir, 01330 Adana, Turkey; ^2^Department of Pathology, Cukurova University, 01330 Adana, Turkey

## Abstract

*Purpose*. To report an unusual case of severe bilateral fungal keratitis following laser in situ keratomileusis (LASIK). *Method*. A 48-year-old man developed bilateral diffuse corneal infiltration two weeks after LASIK. The corneal scrapings revealed fungal filaments but cultures were negative. *Results*. The corneal ulceration was improved on the left eye whereas spontaneous perforation occurred and finally evisceration was needed on the right eye despite topical and systemic antifungal treatment. *Conclusions*. Fungal keratitis, especially with bilateral involvement, is a very rare and serious complication of LASIK surgery. Clinical suspicion is crucial because most of fungal keratitis are misdiagnosed as bacterial keratitis and can lead serious visual results, even eye loss.

## 1. Introduction

Laser in situ keratomileusis is a commonly used and mostly uneventful refractive surgery procedure. Because it is performed in partially aseptic conditions, infection after laser in situ keratomileusis is rare but it is still a sight-threatening, even a blinding complication. The incidence of postoperative infection after LASIK is 1 : 3000 to 1 : 5000 [1]. Postoperative intensive steroid use increases host susceptibility to microorganisms and can lead to opportunistic infections. Various infections of bacterial origin have been described. In this paper, we present a case of severe bilateral fungal keratitis after LASIK.

## 2. Case Report

A 48-year-old man with no significant ocular and medical history had bilateral simultaneous LASIK for −2.50 D myopia at a refractive surgery center. Two weeks after the operation, the patient complained of bilaterally reduction in vision, foreign body sensation, intense pain, and watering. He was diagnosed as having infectious keratitis by his ophthalmologist, who prescribed topical fortified cefazolin, fortified gentamicin every hour, and cyclopentolate 1% three times daily. Upon detection of fungal filaments on the corneal scraping material, the treatment protocol was changed to topical amphotericin B 0,15%, natamycine 5%, and oral ketoconazole. The patient was subsequently referred to our clinic. 

**Figure 1:**
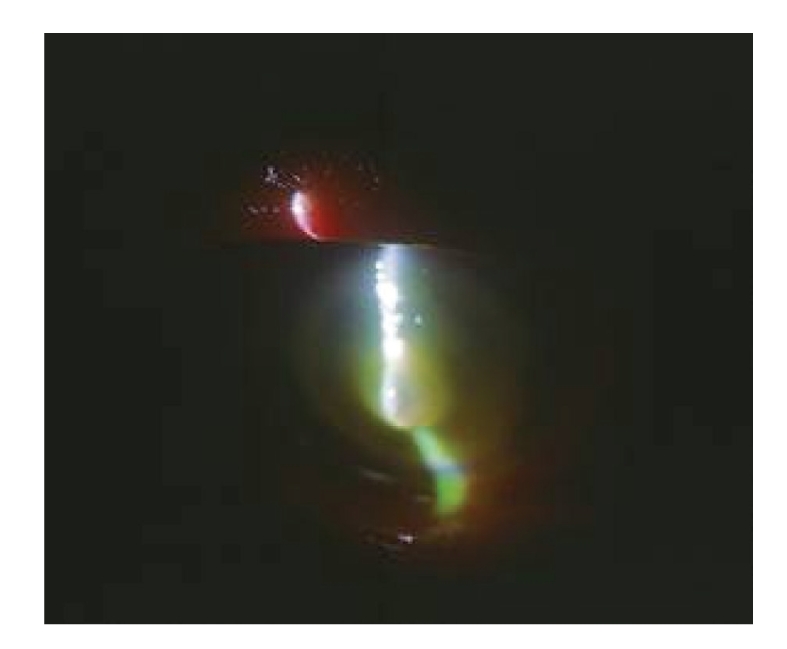
Clinical picture of the right eye at initial presentation. There is marked corneal infiltration, and central thinning.

**Figure 2:**
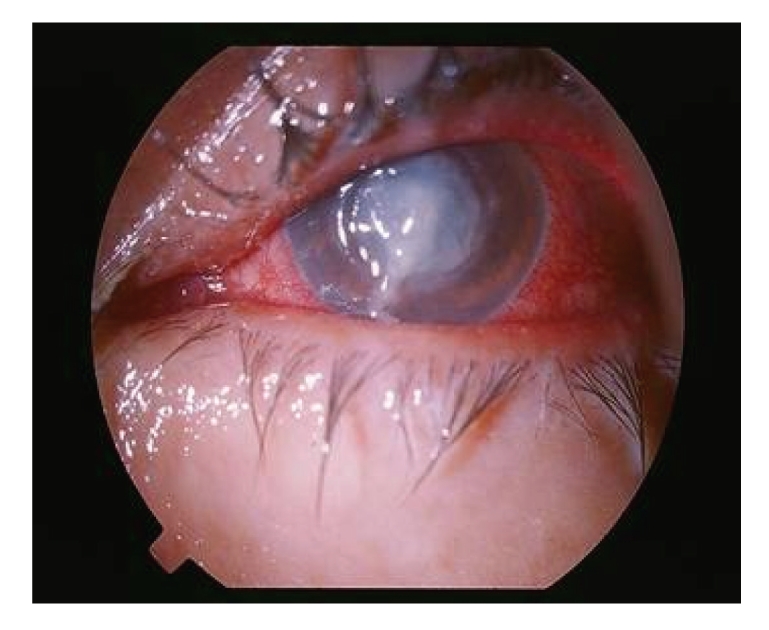
Conjunctival injection, diffuse central corneal infiltration and marked discharge in the left eye at initial presentation.

At the time of presentation, the visual acuities were hand motions bilaterally. He had bilateral intense eyelid edema and deep episcleral injection. Slit lamp examination revealed diffuse full-thickness corneal infiltration with overlying epithelial ulcer on both eyes. (Figures 1 and 2) His right eye had excessive central corneal thinning and 2,5 mm of hypopyon. Details of the anterior chamber, iris, and lens were not detectable in neither eye. 

Fundus examinations could not be performed. B-scan ultrasonography showed no vitreoretinal pathology. Digitally measured intraocular pressures were estimated within normal limits bilaterally. 

**Figure 3:**
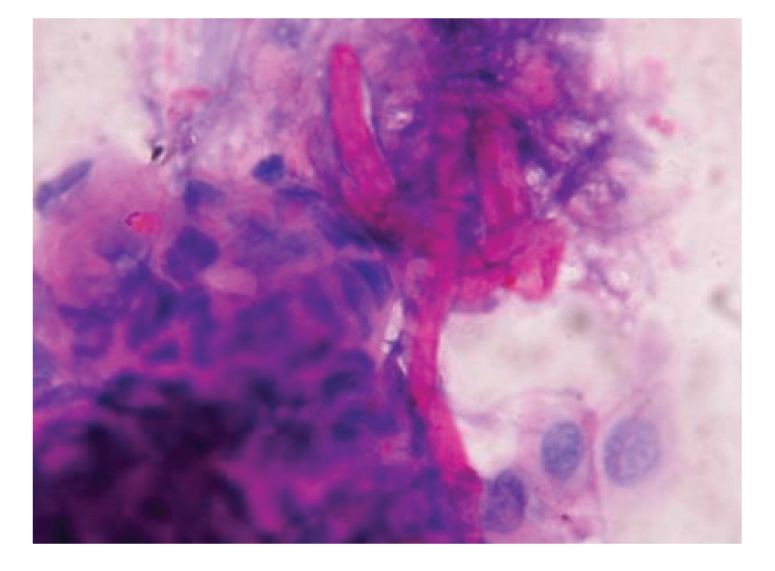
PAS positive fungal hyphae between corneal epithelial cells (×1000).

Corneal scrapings were taken from ulcer beds and edges of both eyes. The microscopic examination of smears with Periodic Acsid Schiff (PAS) stain revealed many fungal filaments (Figure 3). There was no growth on fungal and bacterial culture medias including blood agar, chocolate agar, and Sabouraud dextrose agar. Topical amphotericin B 0,15% and natamycine 5% every hour, cyclopentolate 1% three times daily, and oral ketoconazole 2 × 200 mg twice daily were continued.

**Figure 4:**
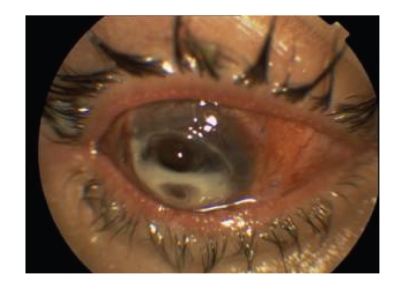
Clinical picture of the right eye after the first amniotic membrane transplantation. There is marked corneal thinning and vascularization.

**Figure 5:**
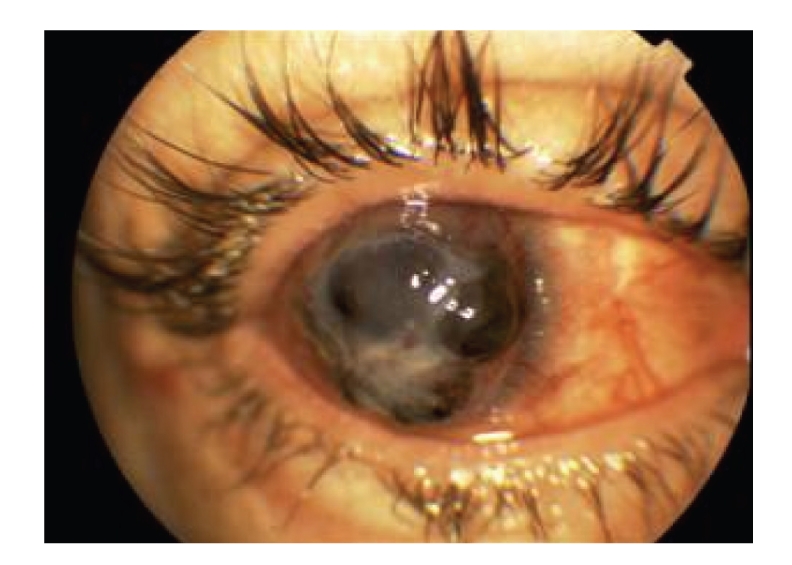
Severe iris prolapse and corneal melting of the right eye after second amniotic membrane transplantation.

Despite antifungal treatment, the clinical appearance worsened. Excessive corneal thinning required repeated amniotic membrane transplantation (Figures 4 and 5). After the second transplantation, spontaneous perforation and iris prolapse occurred on the right eye, visual acuity dropped to no light perception. Eventually, evisceration was performed on that eye. 

**Figure 6:**
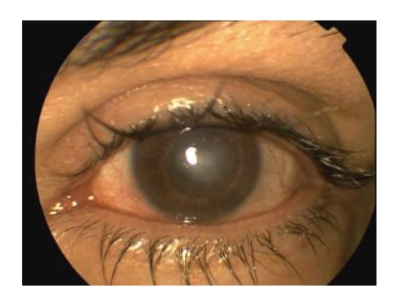
Clinical picture of the left eye 7 months after LASIK. There is minimal conjunctival injection and vascularized corneal leukoma.

During followup, the visual acuity of the left eye improved to counting fingers and the active inflammation subsided (Figure 6). 

## 3. Discussion

LASIK is the most widely used refractive surgery procedure. Although bacterial and fungal keratitis, even endophthalmitis were reported as a complication [2, 3], infections are rare after this extraocular intervention. Moshirfar et al. found that the combined occurrence rate of infectious and noninfectious keratitis after LASIK was 2.66% [4]. Bilateral fungal infection is extremely rare after LASIK [5].

We report here a very severe case of bilateral fungal keratitis after LASIK resulted in evisceration in one eye and severe loss of vision in the other. The fungal pathogen could not be specified by culture, but fungal hyphae were shown on microscopic examination.

Microbial contamination from eyelids, eyelashes, conjunctiva, microkeratome, operative drapes, gloves, and room air is possible during LASIK [6, 7]. Epithelial break after LASIK could allow microbial penetration [8], as well as long-term use of steroids could increase incidence of infection. 

Khan et al. [9] found 5% microbial growth from cultured microkeratome blades used during uncomplicated LASIK procedures. All of the microorganisms were Staphylococcus species sensitive to fourth generation flouroquinolones and vancomycin. None of the cases demonstrated signs of corneal infection. They concluded that antibiotic prophylaxis could have a role to eradicate subclinical bacterial stains. 

Postoperative close follow-up after LASIK is essential because microbial keratitis can occur within days, weeks or even years after LASIK [10]. Vieira and Pereira described late-onset infectious keratitis after LASIK caused by Pseudomonas and Fusarium species [11]. 

The distinction between infection and postoperative inflammatory response must be made carefully for critical therapeutic intervention. Sterile infiltrates require topical corticosteroids, but misdiagnosis of the infection as an inflammation can exacerbate the existing clinical condition and worsen the prognosis.

Various fungal organisms including Candida [12], Fusarium [4], and Alternaria species [13] were isolated from post-LASIK corneas. Aspergillus species [14–16] are the most common cause of fungal keratitis. Most of fungal keratitis are misdiagnosed as bacterial keratitis. The treatment of fungal keratitis is crucial because of the lack of effective, strongly penetrating antifungal agents [17].

 When post-LASIK infection is suspected, microbiological workup should be done immediately for proper treatment because fungal infections progress rapidly; however, culture confirmation and agent identification could not be always obtained [18]. Appropriate treatment is usually delayed because of negative and time-consuming cultures. In this condition, microscopic examination could be helpful in guiding the diagnosis and the decision of antifungal treatment, and also empirical treatment with broad spectrum fortified antibiotics is warranted. Corneal scrapings and conjunctival swabs must be taken. Deep corneal infiltration, Descemet's membrane penetration can occur in fungal corneal infections [19]. For resistant cases, flap lifting, irrigation with antibiotics, daily debridement, and repositioning should be done early. In case of progression and poor response to therapy, flap amputation is also indicated. The flap amputation could no be performed in the present case because of severe thinning and melting of the affected cornea at the time of presentation to our clinic. The progression was so fast that there was not enough time to change the treatment protocol and to switch it to voriconasole. The patient had clinical stability after the amniotic membrane transplantation, the active infection subsided, and so the time of keratoplasty was postponed. The culture negativity was thought to be caused by the time delay and the treatment given between the first presentation and the referral. The fungal cultures were reconfirmed but were still negative. 

Wide use of the LASIK procedure causes inevitable increase in complications, so the reports of post-LASIK infections increased in recent years [20]. Llovet et al., found that the incidence was 0,035% per procedure and detected the following risk factors: blepharitis, intraoperative epithelial defect, dry eye, and the health care environment [21]. Careful attention to patient selection, sterile technique, monitoring the intraoperative and postoperative environmental conditions, and hygiene are important. If bilateral simultaneous surgery is to be performed, change of the lid speculum and the microkeratome blade is mandatory to prevent contamination from the first eye. Clinical suspicion and looking up for preliminary infectious signs are essential in early intervention of antimicrobial therapy. Fungal infections are rare, and antifungal prophylaxis is not rationalized after the procedure. The reason of the fungal infection in the present case was thought to be the inappropriate sterilization of the operating room. However, in case of fungal contaminations, treatment is very difficult due to the difficulty in isolating the microorganism and the lack of very effective antifungal agents for the cornea. 

Some of these cases could not be prevented from the poor prognosis leading to serious sequelae, poor final visual performance, even loss of the eye, as in our case. Patients should be aware of this rare but possible infections and especially possible bilateral complications after LASIK.
